# DNA Methylation Reprogramming during Mammalian Development

**DOI:** 10.3390/genes10040257

**Published:** 2019-03-29

**Authors:** Yang Zeng, Taiping Chen

**Affiliations:** 1 Department of Epigenetics and Molecular Carcinogenesis, The University of Texas MD Anderson Cancer Center, 1808 Park Road 1C, Smithville, TX 78957, USA; yzeng1@mdanderson.org; 2 Center for Cancer Epigenetics, The University of Texas MD Anderson Cancer Center, Smithville, TX 78957, USA; 3 Program in Genetics and Epigenetics, The University of Texas MD Anderson Cancer Center UTHealth Graduate School of Biomedical Sciences, Houston, TX 77030, USA

**Keywords:** DNA methylation, embryogenesis, germ cells, DNMTs, TETs

## Abstract

DNA methylation (5-methylcytosine, 5mC) is a major form of DNA modification in the mammalian genome that plays critical roles in chromatin structure and gene expression. In general, DNA methylation is stably maintained in somatic tissues. However, DNA methylation patterns and levels show dynamic changes during development. Specifically, the genome undergoes two waves of global demethylation and remethylation for the purpose of producing the next generation. The first wave occurs in the germline, initiated with the erasure of global methylation in primordial germ cells (PGCs) and completed with the establishment of sex-specific methylation patterns during later stages of germ cell development. The second wave occurs after fertilization, including the erasure of most methylation marks inherited from the gametes and the subsequent establishment of the embryonic methylation pattern. The two waves of DNA methylation reprogramming involve both distinct and shared mechanisms. In this review article, we provide an overview of the key reprogramming events, focusing on the important players in these processes, including DNA methyltransferases (DNMTs) and ten-eleven translocation (TET) family of 5mC dioxygenases.

## 1. Introduction

DNA Methylation—the addition of a methyl group to the 5-position of cytosine, forming 5-methylcytosine (5mC)—is a major form of DNA modification in many, but not all, eukaryotic organisms. In mammals, DNA methylation mainly occurs in the context of CpG dinucleotides, forming a symmetrical pattern on both strands. In the mouse genome, the majority (~60–80%) of CpG dinucleotides are methylated. Non-CpG (i.e., CpA, CpT, or CpC) methylation is rare except in special cell types, such as embryonic stem cells (ESCs), oocytes, and neurons [1]. 5mC distribution in the genome is bimodal. In general, repetitive sequences, such as transposons and centromeric and pericentric repeats, are heavily methylated, gene bodies of highly expressed genes are also methylated, whereas CpG islands (CGIs), i.e., 500–2000-bp GC-rich sequences that are frequently present in promoter regions, are usually devoid of methylation [2]. DNA methylation is essential for mammalian development and is involved in a variety of biological processes, including transcriptional regulation, transposon silencing, X chromosome inactivation, and genomic imprinting [3]. Aberrant DNA methylation patterns and mutations of genes encoding DNA methylation enzymes or regulators are associated with developmental disorders and cancer [4,5].

**Figure 1:**
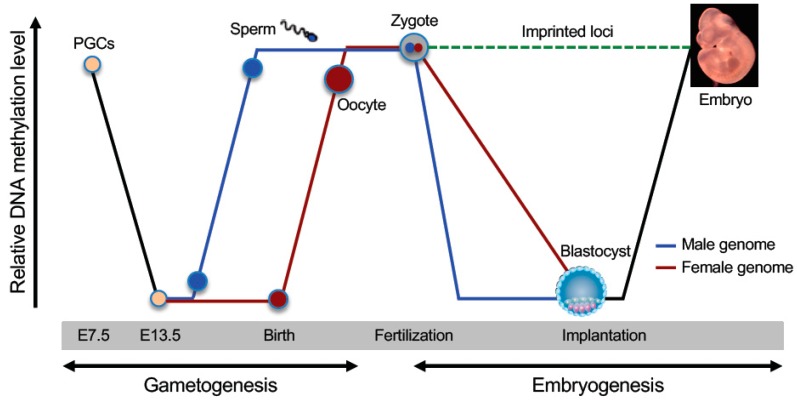
Dynamic changes in DNA methylation during mammalian development. Schematically shown are the two waves of global DNA demethylation and remethylation in the life cycle (adapted from [6]). Primordial germ cells (PGCs) initially have high levels of DNA methylation. Global demethylation occurs during PGC expansion and migration. At later stages of germ cell development (before birth in male and after birth in female), de novo methylation results in the establishment of sex-specific germ cell methylation patterns, including methylation marks at imprinted loci. Shortly after fertilization, the methylation marks inherited from the gametes are erased again (except those at imprinted loci and some retrotransposons), with the paternal genome undergoing active demethylation and the maternal genome undergoing passive demethylation. Upon implantation, a wave of de novo methylation establishes the initial embryonic methylation pattern.

As a stable epigenetic mark that is heritable through cell division, DNA methylation is an important component of the cellular memory mechanism that maintains cell identities. However, the epigenome, including DNA methylation, needs to be reprogrammed to a totipotent state for producing the next generation. There are two waves of global demethylation and remethylation during the mammalian life cycle (Figure 1), one occurring during germ cell development and the other occurring during early embryogenesis [6,7]. Epigenetic reprogramming in the germline involves the erasure of somatic methylation patterns in primordial germ cells (PGCs) and subsequent establishment of sex-specific germ cell methylation patterns, including methylation marks in imprinting control regions (ICRs). Epigenetic reprogramming in early embryos involves erasure of most methylation marks inherited from the gametes (exceptions include methylation marks in ICRs and some retrotransponsons) at preimplantation stages and reestablishment of global DNA methylation patterns upon implantation. In this review, we discuss the highly dynamic and regulated processes of DNA demethylation and remethylation, focusing on the important enzymes and regulators that are involved in these reprogramming events.

## 2. DNA Methyltransferases and Regulators

As CpG/CpG dyads are symmetrical, Holliday and Pugh, and Riggs independently proposed that methylated CpG sites could be heritable through semi-conservative DNA replication [8,9]. The theory would predict at least two catalytic activities—de novo methylation activity for the establishment of methylation patterns and maintenance methylation activity for converting hemi-methylated CpG sites to fully methylated ones during DNA replication. Subsequent work identified DNA methyltransferases (DNMTs) with distinct properties and their accessory factors, including ubiquitin−like with plant homeodomain (PHD) and really interesting new gene (RING) finger domains 1 (UHRF1) and DNMT3L [10].

### 2.1. DNMT1 and UHRF1—Key Components of Maintenance Methylation Machinery

*Dnmt1*, the first mammalian DNA methyltransferase gene discovered, was cloned from murine cells [11]. It has several transcription start sites, producing three major isoforms [12,13]. *Dnmt1s*, expressed in somatic cells, encodes the full-length DNMT1 protein of 1620 amino acids. *Dnmt1o*, specifically expressed in oocytes, encodes a protein product that lacks the N-terminal 118 amino acids due to translation from a downstream start codon. Compared to DNMT1, DNMT1o has similar catalytic activity but appears to be more stable [14]. *Dnmt1p*, specifically expressed in pachytene spermatocytes, does not produce a protein product. Human DNMT1, with full-length protein consisting of 1616 amino acids, shows ~80% sequence identity to murine DNMT1.

**Figure 2:**
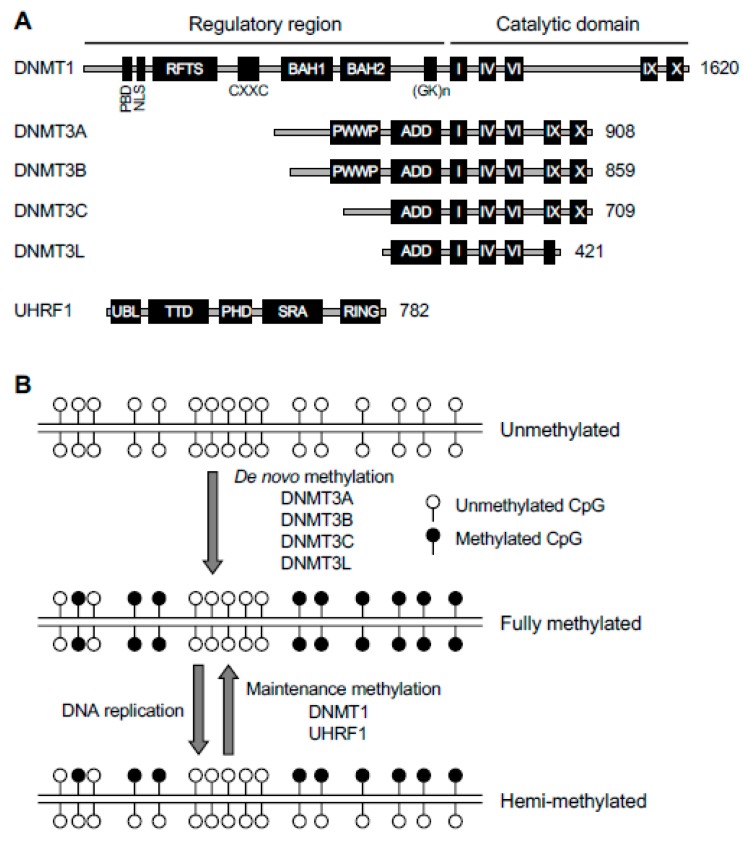
DNA methylation machinery. (**A**) The protein domains in DNA methyltransferases (DNMTs) and ubiquitin−like with plant homeodomain (PHD) and really interesting new gene (RING) finger domains 1 (UHRF1) are shown (the sizes/amino acid numbers refer to mouse proteins). The DNMT1 and DNMT3 families of proteins share conserved catalytic motifs (I–X) in the C-terminal catalytic domains (DNMT3L lacks catalytic activity because some essential motifs are missing or mutated) but have distinct N-terminal regulatory regions. PBD, proliferating cell nuclear antigen (PCNA)-binding domain; NLS, nuclear localization signal; RFTS, replication foci targeting sequence; CXXC, a cysteine-rich zinc finger domain; BAH, bromo-adjacent homology domain; (GK)n, glycine/lysine repeats; PWWP, proline-tryptophan-tryptophan-proline domain; ADD, ATRX-DNMT3-DNMT3L domain; UBL, ubiquitin-like domain; TTD, tandem Tudor domain; PHD, plant homeodomain; SRA, Su(var)3-9, Enhancer of zeste, and Trithorax (SET)- and RING-associated domain; RING, really interesting new gene domain. (**B**) De novo and maintenance methylation activities. The de novo methyltransferases (DNMT3A, DNMT3B, and DNMT3C), in complex with their accessory factor DNMT3L, methylate unmethylated CpG sites to establish methylation patterns. The maintenance methyltransferase DNMT1, in complex with its accessory factor UHRF1, methylates hemi-methylated CpG sites at each round of DNA replication to maintain methylation patterns.

DNMT1 contains a C-terminal catalytic domain that harbors specific motifs (I-X) characteristic of all DNA methyltransferases and a unique N-terminal regulatory domain that harbors several functional domains. These domains include a nuclear localization signal (NLS) that imports DNMT1 to the nucleus, a proliferating cell nuclear antigen (PCNA) binding domain (PBD) that interacts with the DNA replication machinery, a replication foci-targeting sequence (RFTS) that targets DNMT1 to DNA replication foci, a CXXC zinc finger domain that binds unmethylated DNA, and two bromo-adjacent homology (BAH) domains and a glycine-lysine (GK) repeat that are required for the catalytic activity of DNMT1 (Figure 2A). Crystallography data reveal that, in the absence of DNA substrates, the RFTS blocks the catalytic center, suggesting that the N-terminal regulatory region plays a self-inhibitory role for DNMT1 activity [15].

In vitro biochemical assays indicate that DNMT1 prefers DNA substrates containing hemi-methylated CpG sites [16]. *Dnmt1* expression is activated by cell cycle-dependent transcription factors and thus is present at high levels in proliferating cells [17]. Immunofluorescence experiments show enrichment of DNMT1 in replication foci during S phase of the cell cycle, suggesting that its function is coupled with DNA replication [18]. Genetic disruption of *Dnmt1* in mouse ESCs (mESCs) results in global loss of methylation [19,20]. These lines of evidence suggest that DNMT1 plays a major role in maintaining DNA methylation through cell division (Figure 2B). DNMT1 also has de novo methylation activity, which is likely repressed in cells. For example, a recent study suggests that DNMT1-mediated de novo methylation contributes to abnormal hypermethylation in mouse oocytes deficient for the maternal factor STELLA (also known as PGC7 and DPPA3) [21].

UHRF1, also known as NP95 (nuclear protein of 95 kDa) in mouse or ICBP90 (inverted CCAAT box−binding protein of 90 kDa) in human, is an essential accessory factor of DNMT1. *Uhrf1* knockout (KO) mESCs exhibit global hypomethylation [22,23], similar to the phenotype of *Dnmt1* KO mESCs [19,20]. UHRF1 and DNMT1 form a complex and colocalize in replication foci and heterochromatin, and in the absence of UHRF1, DNMT1 fails to localize to these regions [22,23,24]. These findings suggest that UHRF1 is critical in recruiting DNMT1 to hemi-methylated CpG sites during DNA replication (Figure 2B).

UHRF1 contains five conserved domains, all of which have been implicated in DNMT1 function (Figure 2A). The Su(var)3-9, Enhancer of zeste, and Trithorax (SET)- and RING-associated (SRA) domain preferentially binds hemi-methylated DNA and plays an important role in loading DNMT1 onto newly synthesized DNA [22,23]. The tandem Tudor domain (TTD) and the plant homeodomain (PHD) cooperatively interact with the N-terminal tail of histone H3 by recognizing a specific histone modification signature consisting of trimethylated Lys9 (H3K9me3) and unmethylated Arg2 (H3R2me0) [24,25,26,27,28,29]. The really interesting new gene (RING) domain has E3 ubiquitin ligase activity and monoubiquitinates several lysine residues in the N-terminal tail of histone H3, creating DNMT1-binding sites [30,31,32]. There is evidence that SRA domain-mediated recognition of hemi-methylated DNA allosterically activates RING domain-mediated H3 ubiquitination and facilitates histone binding [32,33]. Recently, the ubiquitin-like (UBL) domain was shown to interact with the E2 ubiquitin conjugating enzyme UBE2D to facilitate H3 monoubiquitination [34,35]. Thus, UHRF1 acts as both a ‘writer’ and ‘reader’ of histone marks and targets DNMT1 to its DNA substrates via complex, multivalent interactions with chromatin.

### 2.2. DNMT3A, DNMT3B, DNMT3C, and DNMT3L—Key Components of de novo Methylation Machinery

By homology searches using bacterial DNA methyltransferase genes, Okano et al. identified two mammalian homologues, *Dnmt3a* and *Dnmt3b* [36]. *Dnmt3a* has two major promoters, producing the *Dnmt3a1* and *Dnmt3a2* isoforms. *Dnmt3a1* encodes the full-length DNMT3A protein (908 amino acids in mouse and 912 amino acids in human), and *Dnmt3a2*, driven by a downstream intronic promoter, encodes a protein product that lacks the N-terminal 219 (mouse) or 223 (human) amino acids. DNMT3A and DNMT3A2 have similar catalytic activities [37]. Due to alternative splicing, *Dnmt3b* produces multiple isoforms, with some encoding active enzymes (e.g., full-length DNMT3B1, 859 amino acids in mouse and 853 amino acids in human) and others encoding catalytically inactive protein products. Some inactive DNMT3B proteins function as regulators of DNA methylation [38,39].

DNMT3A and DNMT3B have similar structures (Figure 2A), including a C-terminal catalytic domain with the conserved catalytic motifs and an N-terminal regulatory domain with no sequence similarity to DNMT1. The N-terminal region contains two chromatin-interaction domains that likely play important roles in determining the functional specificity of DNMT3A and DNMT3B: A proline-tryptophan-tryptophan-proline (PWWP) domain that recognizes H3K36me3 via an aromatic cage and also binds DNA through a positively charged surface [40,41,42,43,44] and an ATRX-DNMT3-DNMT3L (ADD) domain that interacts with the histone H3 tail with H3K4me0 [45]. Crystal structure indicates that the ADD domain of DNMT3A interacts with its own catalytic domain to inhibit enzymatic activity, resulting in autoinhibition. H3K4me0 peptide, but not H3K4me3 peptide, can disrupt the interaction between the ADD and catalytic domains and thus releases the autoinhibition [46], suggesting that histone marks not only create binding sites for DNMT3A/3B but also stimulate their catalytic activity.

Based on several lines of evidence, DNMT3A and DNMT3B primarily carry out de novo DNA methylation (Figure 2B). First, their expression correlates with de novo methylation during development. Specifically, *Dnmt3a* (mainly *Dnmt3a2*) and *Dnmt3b* (mainly *Dnmt3b1*) are highly expressed in early embryos (as well as mESCs) and are downregulated in differentiated cells (mainly *Dnmt3a1*, *Dnmt3b2,* and *Dnmt3b3*), and *Dnmt3a* (mainly *Dnmt3a2*) is also abundantly expressed in developing germ cells [36,47,48,49]. Second, in vitro assays indicate that DNMT3A and DNMT3B methylate DNA substrates containing unmethylated and hemi-methylated CpG sites with equal efficiency [36]. Third, acute deletion of *Dnmt3a* and *Dnmt3b* in mESCs does not affect global methylation but prevents de novo methylation of newly integrated proviruses [50]. It is worth mentioning that *Dnmt3a/3b* double KO mESCs show gradual loss of methylation with continuous culturing [51], suggesting that, while DNMT1 is the major maintenance enzyme, DNMT3A and DNMT3B are also required for stable and faithful maintenance of DNA methylation.

*Dnmt3c*, a *Dnmt3b* duplicated gene present only in rodent genomes, was initially identified as a pseudogene for lack of expression and recognizable open reading frame [52]. Recent work showed that *Dnmt3c* is expressed during spermatogenesis, encoding a protein of 709 amino acids that is similar to DNMT3B but lacks the PWWP domain in the N-terminal regulatory region. Genetic evidence demonstrates that *Dnmt3c* is not required for mouse development but is essential for normal spermatogenesis by methylating and silencing special transposons in the male germline [53].

*Dnmt3l*, another member of the *Dnmt3* family, encodes a catalytically inactive protein that contains some, but not all, catalytic motifs in the C-terminal region and an ADD domain, but not a PWWP domain, in the N-terminal region [54,55,56]. Crystal structure reveals that DNMT3A and DNMT3L form a tetramer, with two DNMT3A molecules in the middle and DNMT3L on each side [57,58]. DNMT3L substantially stimulates the catalytic activity of DNMT3A and DNMT3B in vitro [59,60,61]. Recent work suggests that DNMT3L is also involved in maintaining DNMT3A stability [62]. In addition, the ADD domain of DNMT3L binds H3K4me0 and likely plays a role in determining the specificity of DNMT3A-mediated DNA methylation [63]. These results indicate that DNMT3L is an important accessory factor of DNMT3A (and perhaps DNMT3B as well) (Figure 2B). Contrary to previous evidence that DNMT3L is a positive regulator [56,59,60,61,64,65,66,67], Neri et al. reported gain of methylation at bivalent promoters in *Dnmt3l* knockdown (KD) mESCs and concluded that DNMT3L antagonizes DNA methylation at some genomic regions [68]. However, genome-wide analysis of *Dnmt3l*-deficient mESCs revealed only loss of methylation, mostly at DNMT3A target loci, in a recent study [62], disputing the finding and conclusion of the Neri paper [68].

### 2.3. DNMT2/TRDMT1

Another protein, initially named DNMT2, contains the catalytic motifs characteristic of DNMTs but is not required for either de novo or maintenance DNA methylation [69]. Subsequent work shows that it methylates aspartic acid tRNA at cytosine 38. Thus, it has been renamed tRNA aspartic acid [D] methyltransferase 1 (TRDMT1) [70]. While *Dnmt2* KO mice are viable and grossly normal [69], recent evidence suggests that DNMT2/TRDMT1-dependent RNA modifications play an important role in intergenerational transmission of paternally acquired metabolic disorders to offspring by regulating the sperm small RNA expression profile, including tRNA- and rRNA- derived small RNAs [71].

## 3. DNA Demethylation

Although DNA methylation is generally stable in somatic tissues, loss of methylation occurs during development, cellular differentiation, aging, and in cancer cells. In general, loss of methylation can be achieved by two mechanisms: DNA replication-dependent passive dilution of 5mC and enzyme-mediated active removal/replacement of 5mC [72].

### 3.1. Passive DNA Demethylation

During DNA replication, methylation patterns are maintained by the maintenance methylation machinery, including DNMT1 and its accessory factor UHRF1. Therefore, functional deficiency in maintenance methylation can lead to replication-dependent dilution of 5mC, known as passive DNA demethylation. This mechanism, which allows efficient erasure of global DNA methylation, is relevant in multiple biological processes. In preimplantation embryos, erasure of DNA methylation marks inherited from the oocyte (i.e., in the maternal genome) is mainly through passive demethylation during cleavage division, presumably due to the exclusion of DNMT1 from the nuclei [6,7]. In PGCs, the first phase of global demethylation is a passive process, as a result of the silencing of several key DNA methylation enzymes and regulators, including UHRF1 [73]. In mESC culture, there exists a small population of totipotent cells, similar to 2-cell (2C) embryos, at any given time. It is believed that mESCs convert to the transient 2C-like state to recover shortened telomeres and repair DNA damage. Recent work reveals that, due to UHRF1 and DNMT1 degradation, 2C-like cells are severely hypomethylated, which is required for telomere elongation [74]. Cancer cells generally exhibit global loss of methylation and regional gain of methylation. While the mechanisms underlying these aberrant changes are complex, overexpression of PRMT6, an enzyme that deposits asymmetric dimethylation of H3R2 (H3R2me2a), was recently shown to contribute to global hypomethylation in cancer cells, as H3R2me2a inhibits the recruitment of the UHRF1-DNMT1 complex to chromatin [75].

### 3.2. Active DNA Demethylation

Rapid loss of DNA methylation marks can also occur in slowly or non-dividing cells, which cannot be explained by passive demethylation. As the methyl group in 5mC is linked to cytosine by a carbon-carbon bond, demethylation by direct cleavage of the methyl group is considered infeasible. Active DNA demethylation, which is independent of DNA replication, refers to processes that result in indirect removal of 5mC, involving enzyme-mediated modifications of 5mC followed by replacement with unmodified cytosine. Significant progress has been made in understanding the mechanism of active demethylation since the discovery that 5mC can be converted to oxidized forms by the ten-eleven translocation (TET) enzymes [76,77].

**Figure 3:**
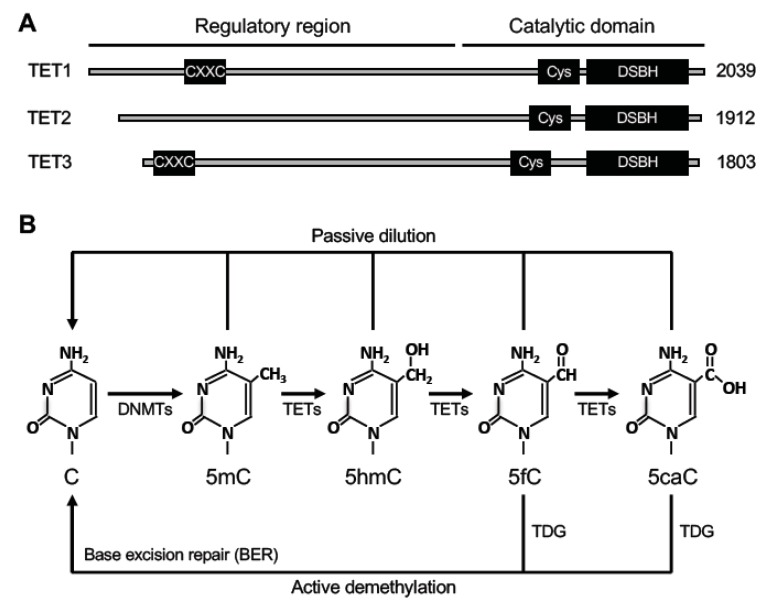
Ten-eleven translocation (TET) proteins and relevant DNA demethylation pathways. (**A**) The protein domains in TET proteins (TET1, TET2 and TET3) are shown (the sizes/amino acid numbers refer to mouse proteins). Their C-terminal catalytic domains contain two characteristic sequence features, a cysteine-rich region (Cys) and a double-stranded β helix (DSBH) fold. Their N-terminal regulatory regions are less conserved, with TET1 and TET3 containing a CXXC zinc finger domain. (**B**) DNA demethylation pathways involving TETs. TET proteins initiate DNA demethylation by oxidizing 5-methylcytosine (5mC) to 5-hydroxymethylcytosine (5hmC), which can be further oxidized to 5-formylcytosine (5fC) and 5-carboxylcytosine (5caC). 5fC and 5caC can be excised by thymine DNA glycosylase (TDG). The residual abasic site can then be repaired by the base excision repair (BER) pathway to complete ‘active’ demethylation. 5mC, 5hmC, 5fC, and 5caC can also be removed through DNA replication-coupled ‘passive’ dilution.

There are three members in the TET family, TET1, TET2, and TET3 (Figure 3A). They are capable of oxidizing 5mC into 5-hydroxymethylcytosine (5hmC), 5-formylcytosine (5fC), and 5-carboxylcytosine (5caC) [76,77,78,79]. All three TET proteins have similar structures, with a C-terminal catalytic domain and an N-terminal regulatory domain. The C-terminal catalytic domain contains a cysteine-rich region (Cys) and a double-stranded β-helix (DSBH) domain. The catalytic core region preferentially recognizes 5mC as a substrate and also binds to 5mC oxidized derivatives [80]. TET1 and TET3, but not TET2, contain a DNA-binding CXXC zinc finger domain in the N-terminal regulatory domain. During evolution, the ancestral *Tet2* gene was split into two distinct genes, *Idax* and the current *Tet2*. IDAX, which contains a CXXC domain, interacts with TET2, and may be involved in recruiting TET2 to its genomic targets [81].

Studies over the last decade have demonstrated that 5hmC, 5fC, and 5caC serve as intermediates of DNA demethylation (Figure 3B). 5fC and 5caC can be recognized and excised from DNA by thymine DNA glycosylase (TDG) [79,82,83]. The residual abasic site can then be repaired by the base excision repair (BER) pathway. Thus, this process results in the replacement of 5mC by an unmodified cytosine, achieving ‘active’ demethylation. It is important to point out that passive dilution is also involved in TET-mediated DNA demethylation. Since 5mC oxidized derivatives are poorly recognized by DNMT1 [84], these marks could be lost owing to the lack of maintenance during DNA replication.

## 4. DNA Methylation Reprogramming in the Germline

In early postimplantation embryos, a small number of cells are set aside as PGCs that eventually develop into germ cells (sperm or oocyte) for reproduction. Epigenetic reprogramming in the germline involves the erasure of somatic lineage epigenetic marks (including DNA methylation) in PGCs and subsequent establishment of germ cell-specific epigenomes.

### 4.1. Biphasic Demethylation in PGCs

In mice, PGCs are specified from the posterior epiblasts at the onset of gastrulation around E6.25-7.25, in response to instructive signals from extraembryonic tissues, including the transcription factors BLIMP1, PRDM14, and BMP4 signaling [85]. After specification, PGCs migrate from the proximal epiblast along the hindgut (~E7.75) and reach the genital ridges (~E10.5) and subsequently start sex differentiation (~E12.5).

**Figure 4:**
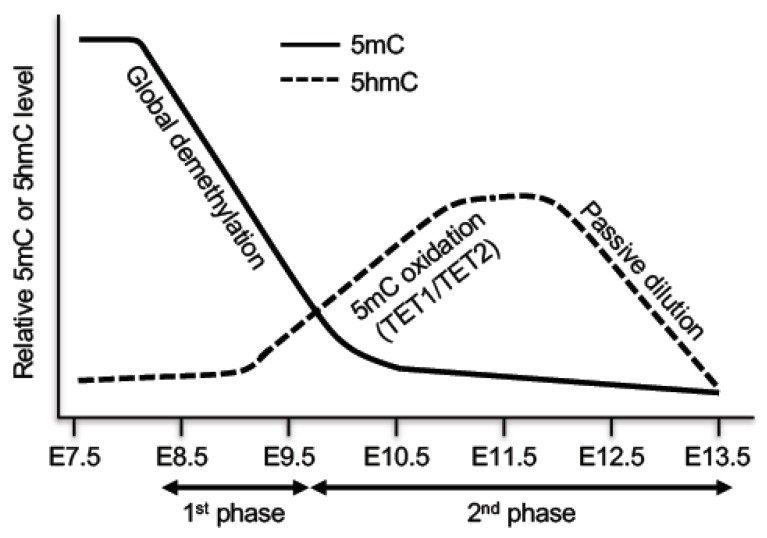
DNA demethylation in primordial germ cells (PGCs). PGCs undergo DNA demethylation in two phases. The first phase is mainly a passive process due to repression of important components of the DNA methylation machinery, resulting in global demethylation. The second phase, which affects specific loci including imprinted genes, is initiated by TET1- and TET2-mediated 5mC oxidation, followed by passive dilution of oxidized derivatives.

As the epiblast rapidly adopts somatic epigenetic features after implantation, PGCs are initially very similar to their somatic counterparts in DNA methylation patterns, transcription profiles, and chromatin modifications, including silencing of pluripotency genes and germline-specific genes by DNA methylation [7]. At this point, PGCs display high levels of 5mC and low levels of 5hmC. In the initial stage of migration at E7.5–E8.5, PGCs arrest in the G2 phase of the cell cycle, then they start rapid proliferation until at least E12.5 [7]. Genome-wide DNA methylation profiling reveals that PGCs undergo DNA demethylation in two distinct phases [86,87] (Figure 4). The first phase, starting at ~E8.5, occurs during PGC proliferation and migration, which results in genome-wide loss of methylation, involving almost all genomic sequences. Passive demethylation is predominantly responsible at this phase, due to deficiency in major components of the methylation machinery. For example, *Uhrf1* is repressed and excluded from the nucleus at E9.5. Only after E12.5, *Uhrf1* expression starts to increase and DNMT1 localization is slowly restored back to replication foci [73]. The second phase occurs from E9.5 to E13.5, which leads to demethylation of specific loci, including imprinted genes, CGIs of the inactive X chromosome in females, and germline-specific and meiosis-specific genes [86,87,88,89]. Active demethylation is mainly responsible for removing methylation marks at these loci, which are protected from passive demethylation in early PGCs. TET1- and TET2-mediated conversion of 5mC to 5hmC plays an important role in the second phase of DNA demethylation. Consistent with the TET1 and TET2 expression patterns, 5hmC level increases in E9.5–E10.5 PGCs with decreasing 5mC levels, peaks at E11.5, and then gradually declines at E11.5–E13.5 [88,89,90,91]. Genetic studies confirm the functions of TET1 in erasure of genomic imprinting and in activation of germline genes involved in gamete generation and meiosis [92,93,94]. Taken together, both passive and active demethylation pathways are involved in global demethylation, including the erasure of parental imprints, in PGCs. At E13.5, PGC genomes display the lowest level of global DNA methylation in the life cycle.

### 4.2. Sex-Specific Remethylation in the Germline

Sex-specific development of the mouse embryo starts at ~E12.5. Following erasure of DNA methylation marks in PGCs, male and female germ cells undergo remethylation at different time points during gametogenesis (Figure 1). While undergoing mitotic expansion in the developing gonad, the male germline starts the remethylation process as early as E14.5, and the methylation patterns are fully established by the time of birth. After sexual maturity, male germ cells undergo many rounds of mitotic proliferation to form spermatocytes before entering meiotic differentiation to form haploid spermatids. The sperm-specific methylation pattern is maintained during mitotic expansion [95]. The female germline undergoes mitotic expansion before primary oocytes arrest in prophase of meiosis I around the time of birth. After sexual maturity, oocytes complete meiosis I and arrest again in metaphase of meiosis II until fertilization. DNA methylation levels in primary oocytes before birth remain low, and remethylation occurs after birth in the oocyte growth phase [95]. In addition to differences in the timing of DNA methylation, the extent and distribution of DNA methylation are widely different between male and female germ cells. Sperm genomes are almost fully methylated (~90% of CpGs) except CGIs, whereas oocyte genomes show lower methylation levels (~40% of CpGs), with methylation marks being largely confined to intragenic regions of active genes [96].

### 4.3. Establishment of Methylation Imprints

Unlike most genes that show biallelic expression, imprinted genes are expressed from only one allele according to the parent of origin. Nearly 200 imprinted genes have been identified in mouse and 165 in human [97]. These genes are involved in many important processes, including embryogenesis, placenta formation, fetal and postnatal growth, and animal behavior [97,98]. Defects in imprinted gene expression are associated with reproductive disorders (e.g., infertility, hydatidiform mole) and congenital diseases (e.g., Prader−Willi syndrome, Angelman syndrome, Beckwith−Wiedemann syndrome, Silver−Russell syndrome) [99]. Loss of imprinting (i.e., biallelic expression or silencing of imprinted genes) is also frequently observed in cancer cells [100].

The majority of imprinted genes exist in clusters. In general, each cluster contains an ICR with one or more differentially methylated regions (DMRs) on the maternal and paternal alleles. These allele-specific methylation imprints are established in germ cells and, after fertilization, they are maintained during embryonic development and in somatic tissues.

As mentioned above, imprinted DMRs are erased in PGCs and subsequently remethylated, along with other genomic sequences, in a sex-specific manner. Most imprints are maternally derived, and their establishment occurs during the oocyte growth period after birth and is largely completed by the germinal vesicle stage of development and resumption of meiosis [66,101,102]. Only four paternally imprinted genes (i.e., *Igf2−H19*, *Dlk1−Gtl2*, *Rasgrf1,* and *Zdbf2*) are known. Paternal methylation imprints are acquired before birth and maintained through many cycles of mitotic division before entry into meiosis [99].

Genetic studies in mice demonstrate that DNMT3A is responsible for de novo methylation in the germline and its accessory factor DNMT3L is also required [56,64,103]. DNMT3A2 is the major isoform in developing germ cells [48,49]. DNMT3L may facilitate DNMT3A2 function by regulating DNMT3A2 enzymatic activity, stability, and targeting to specific genomic regions [59,60,61,62,63]. A proper chromatin environment is important for the DNMT3A2-DNMT3L complex to be recruited to its genomic targets, including imprinted loci. Biochemical and structural evidence indicates that H3K4 methylation disrupts the interaction between the DNMT3L/DNMT3A ADD domains and the N-terminal tail of histone H3 [45,63], which is supported by in vivo data obtained from genetic studies in mice. Oocytes deficient for KDM1B (also known as LSD2 and AOF1), an H3K4me1/2 demethylase, fail to establish imprinted DMRs at some maternally imprinted loci, as well as methylation marks in other regions, leading to a maternal-effect lethal phenotype [104,105]. A single-amino-acid mutation in the DNMT3L ADD domain causes overall reductions in DNA methylation, coupled with spermatogenesis defects [106]. Also, transcription across DMRs may create an open chromatin environment that facilitates imprint establishment in the germline. Truncation of transcript from an upstream promoter results in the absence of maternal methylation of DMRs at the *Gnas* locus in mouse oocytes [107].

## 5. DNA Methylation Reprogramming in Early Embryos

**Figure 5:**
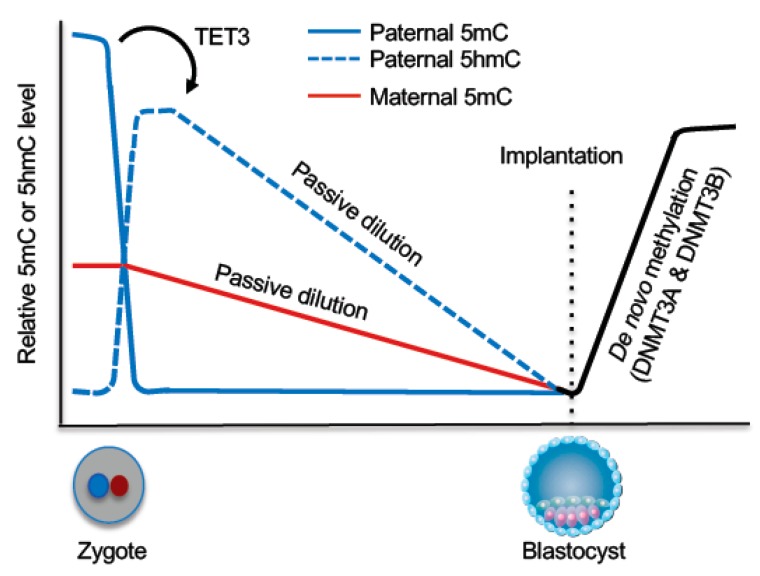
DNA demethylation and remethylation during early embryogenesis. Shortly after fertilization, TET3-mediated 5mC oxidation occurs in the paternal genome and the oxidized derivatives are subsequently removed through passive dilution during preimplantation development. The maternal genome mainly undergoes passive demethylation during preimplantation development. Upon implantation, a wave of de novo methylation establishes the embryonic methylation pattern.

The purpose of epigenetic reprogramming during early embryogenesis is to acquire totipotency and create an epigenome for embryonic development. Key reprogramming events include the erasure of most DNA methylation marks inherited from the gametes in preimplantation embryos and the establishment of embryonic methylation patterns upon implantation (Figure 5).

### 5.1. DNA Demethylation During Preimplantation Development

In the zygote, the parental genomes are spatially separated in maternal and paternal pronuclei, with the maternal genome having a lower level of global DNA methylation than the paternal genome. The parental genomes then undergo genome-wide DNA demethylation with distinct mechanisms. As a consequence, blastocyst-stage embryos reach the lowest methylation levels. However, some regions escape this wave of demethylation, including some retrotransposons and the DMRs of imprinted loci (Figure 5).

Mature sperm genomes have the highest DNA methylation level of all cell types in mammals, with the vast majority (~90%) of CpGs being methylated. Earlier immunofluorescence data showed that the paternal genome loses the 5mC signal before the onset of DNA replication [108,109], suggesting that the paternal genome undergoes active demethylation. Subsequent work demonstrates that 5mC in the paternal genome is converted to 5hmC, 5fC, and 5caC by TET3, which is highly expressed in oocytes and thus abundant in zygotes [110,111]. Rather than being rapidly replaced by unmodified cytosine, 5hmC, 5fC, and 5caC persist in the paternal genome and undergo gradual decline during cleavage divisions [112,113], indicating that, although these 5mC derivatives are produced in the zygote by an enzyme-catalyzed process, their loss during preimplantation development is mainly through a DNA replication-coupled passive process.

The maternal and paternal genomes are exposed to an identical environment in the zygote, yet the maternal genome is protected from TET3-mediated 5mC oxidation. STELLA (also known as DPPA3 and PGC7), a maternal factor, is required for this protection by inhibiting the recruitment of TET3 to the maternal pronucleus [114]. Loss of 5mC in the maternal genome mainly results from DNA replication-dependent dilution during preimplantation development, presumably due to DNMT1 exclusion from the nuclei [115,116]. In preimplantation embryos, DNMT1o, transmitted from oocytes, is the predominant DNMT1 isoform [117], which lacks the N-terminal 118 amino acids of full-length DNMT1. However, the absence of this region does not cause the localization change, as ectopically expressed DNMT1o in somatic cells localizes in the nuclei [115]. The mechanism underlying DNMT1o cytoplasmic localization in early embryos remains to be determined.

### 5.2. Maintenance of Methylation Imprints

Despite the extensive DNA demethylation during preimplantation development, methylation marks in DMRs in imprinted loci are spared. Genetic evidence reveals the requirement of *Dnmt1*, but not *Dnmt3a* and *Dnmt3b*, in the maintenance of methylation imprints [117]. Thus, even though the majority of DNMT1 is excluded from the nucleus, it is not exclusively retained in the cytoplasm. While how the limited amount of DNMT1 is targeted to imprinted loci is not fully understood, the ZFP57-TRIM28 complex plays a critical role. ZFP57 is a Kruppel-like zinc finger protein that binds a consensus hexanucleotide (TGCCGC) present in ICRs, but only when the CpG site is methylated [118]. Removal of ZFP57 from the mouse zygote results in embryonic and neonatal lethality due to defects in the maintenance of both maternal and paternal imprints at multiple imprinted loci [119]. The function of ZFP57 is evolutionarily conserved, as loss-of-function mutations in human are associated with ICR hypomethylation at multiple imprinted loci, resulting in transient neonatal diabetes [120]. TRIM28 (also known as KAP1) is a scaffold protein that assembles a heterochromatin-inducing complex, comprised of SETDB1 (a histone methyltransferase catalyzing H3K9me3), the nucleosome remodeling and histone deacetylation (NuRD) complex, heterochromatin protein 1 (HP1), and the DNA methylation machinery including DNMT1 and UHRF1 [121]. Deletion of *Trim28* in the maternal germ line leads to hypomethylation at several maternal and paternal ICRs and embryonic lethality [121]. In addition to DNMT1-mediated maintenance of methylation marks in ICRs, mechanisms that prevent them from being erased are also important. STELLA has been shown to protect methylation of maternally imprinted genes, as well as two paternally imprinted genes, *H19* and *Rasgrf1* [122], presumably by binding to H3K9me2 to block the recruitment of TET3. During spermatogenesis, the majority of the core histones are replaced by protamines. There is evidence that H3K9me2 is present at the DMRs of *H19* and *Rasgrf1* during protamine exchange [114].

### 5.3. De novo Methylation in the Epiblast

After implantation, a wave of de novo methylation occurs in the epiblast to establish the new methylation pattern in the embryo and start a new life cycle. Genome-wide analysis of mouse embryos from blastocyst to post-implantation stages reveals that DNA methylation is established within two days of implantation between E4.5 and E6.5 [123]. De novo methylation is catalyzed by DNMT3A and DNMT3B. *Dnmt3a* KO mice survive to term but become runted and die within four weeks after birth, inactivation of *Dnmt3b* results in embryonic lethality after E12.5, and embryos deficient for both *Dnmt3a* and *Dnmt3b* exhibit a more severe developmental phenotype and die earlier than *Dnmt3b* KO embryos [50]. In agreement with the developmental phenotypes, DNA methylation analysis of mutant embryos indicates that DNMT3A and DNMT3B act redundantly in methylating the bulk genome and repetitive elements, whereas DNMT3B has a prominent role in methylating CpG islands [123,124]. While what specify DNA methylation patterns is not fully understood, chromatin structure is likely a major determinant. Numerous histone modifications and enzymes/regulators involved in chromatin structure positively or negatively modulate DNA methylation [125,126]. Despite the essential role of DNMT3L in DNA methylation in the germline, it is not required for embryonic development [56,64,65]. There is indication that *Dnmt3l* deficiency causes lower levels of DNA methylation in early embryos, which are subsequently recovered during development [62,127].

## 6. Future Directions

The purpose of the two waves of epigenetic reprogramming is to create appropriate epigenetic states for the initiation and development of the next generation. Among the epigenetic reprogramming events, dynamic changes in DNA methylation, including global erasure and subsequent re-establishment, during germ cell development and early embryogenesis are well documented. Over the last three decades, great progress has been made in understanding the DNA methylation and demethylation machineries, including the identification and characterization of DNMTs, TETs, and key regulators. However, numerous important questions regarding DNA methylation reprogramming remain to be answered. For example, passive demethylation of the maternal genome during preimplantation development is presumably due to exclusion of DNMT1 from the nuclei. Little is known about the mechanisms by which DNMT1 is excluded from the nuclei and/or sequestered in the cytoplasm. Studies in the last several years indicate that demethylation of the paternal genome is initiated by TET3-mediated 5mC oxidation, which is considered an ‘active’ process, but the loss of 5hmC, 5fC, and 5caC is mainly achieved via DNA replication-dependent ‘passive’ dilution. It remains to be determined why these 5mC oxidized derivatives can be maintained at steady-state levels in ESCs and somatic cells, but not in cells of preimplantation embryos. Another issue that is poorly understood is the determinants of sex-specific DNA methylation during germ cell development, including the establishment of methylation imprints. Specifically, it is largely unknown why male and female germ cells are differentially methylated, despite their almost identical genomes (except sex chromosomes). Comprehensive understanding of epigenetic reprogramming in vivo will facilitate development of experimental reprogramming in vitro and could also be valuable for developing novel therapeutic strategies for infertility, imprinting diseases, and other developmental disorders.
